# Crystal structure of a helical silver(I) coordination polymer based on an unsymmetrical dipyridyl ligand: *catena*-poly[[silver(I)-*μ*-*N*-(pyridin-4-ylmeth­yl)pyridine-3-amine-*κ* ^2^ *N*:*N*′] tetra­fluorido­borate methanol hemisolvate]

**DOI:** 10.1107/S205698901501837X

**Published:** 2015-10-07

**Authors:** Suk-Hee Moon, Youngjin Kang, Ki-Min Park

**Affiliations:** a Department of Food and Nutrition, Kyungnam College of Information and, Technology, Busan 47011, South Korea; b Division of Science Education, Kangwon National University, Chuncheon 24341, South Korea; c Research Institute of Natural Science, Gyeongsang National University, Jinju 52828, South Korea

**Keywords:** crystal structure, silver(I) tetra­fluorido­borate, unsymmetrical dipyridyl ligand, helical coordination polymer

## Abstract

The asymmetric unit of the title compound, {[Ag*L*]·BF_4_·0.5CH_3_OH}_*n*_, *L* = *N*-(pyridin-4-ylmeth­yl)pyridine-3-amine, C_11_H_11_N_3_, contains one Ag^I^ ion, one ligand *L*, one tetra­fluorido­borate anion disordered over two orientations in a 0.669 (13):0.331 (13) ratio and one half of a methanol solvent mol­ecule situated on an inversion center. Each Ag^I^ ion is coordinated by two N atoms from two *L* ligands in a distorted linear geometry [N—Ag—N = 174.70 (19)°]. Each *L* ligand bridges two Ag^I^ ions, thus forming polymeric helical chains propagating in [010]. In the crystal, Ag⋯Ag [3.3369 (10) Å] and π–π inter­actions between the aromatic rings [centroid-to-centroid distance = 3.676 (4) Å] link these chains into layers parallel to (10-1). Ag⋯F and weak N(C)—H⋯F inter­actions further consolidate the crystal packing.

## Abstract 2

The reaction of AgBF_4_ with the unsymmetrical ligand *N*-(pyridin-4-ylmeth­yl)pyridine-3-amine afforded a helical coordination polymer. The Ag^I^ atom adopts a slightly distorted linear coordination geometry. The symmetry-related right- and left-handed helical chains are arranged alternately *via* Ag⋯Ag and Ag⋯F inter­actions and π–π stacking inter­actions, resulting in the formation of a two-dimensional supra­molecular network.

## Chemical context   

In supra­molecular chemistry and material science, infinite helical coordination polymers have attracted particular inter­est for the past two decades because of their fascinating architecture, their similarities to biological systems and their potential applications in catalysis and optical materials (Leong & Vittal, 2011 ▸; Wang *et al.*, 2012 ▸; Zhang *et al.*, 2009 ▸). Despite numerous examples of helical coordination polymers, the rational strategy of construction of helical coordination polymers is still constrained by our poor understanding of the role of the metal ions and spacer ligands. Nevertheless, the combination of a silver ion with a linear coordination geometry and flexible unsymmetrical dipyridyl ligands composed of two terminal pyridines with different substituted-nitro­gen positions is one of the most promising strategies for achieving helical coordination polymers. Our group and that of Gao have already reported helical coordination polymers obtained through the reactions of silver salts and some unsymmetrical dipyridyl ligands such as *N*-(pyridin-3-ylmeth­yl)pyridine-2-amine (Moon & Park, 2013 ▸), *N*-(pyridin-2-ylmeth­yl)pyridine-3-amine (Moon & Park, 2014 ▸) and *N*-(pyridin-4-ylmeth­yl)pyridine-3-amine (Moon *et al.*, 2014 ▸; Lee *et al.*, 2015 ▸; Zhang *et al.*, 2013 ▸). Herein, we report the crystal structure of the title compound prepared by the reaction of silver tetra­fluorido­borate with the unsymmetrical dipyridyl ligand, *N*-(pyridin-4-ylmeth­yl)pyridine-3-amine (*L*), synthesized according to the procedure described by Lee *et al.* (2013 ▸). The structure of the title compound is related to those of the Ag^I^ coordination polymers with three different counter-anions such as nitrate, perchlorate and tri­fluoro­methane­sulfonate (Moon *et al.*, 2014 ▸; Lee *et al.*, 2015 ▸; Zhang *et al.*, 2013 ▸).
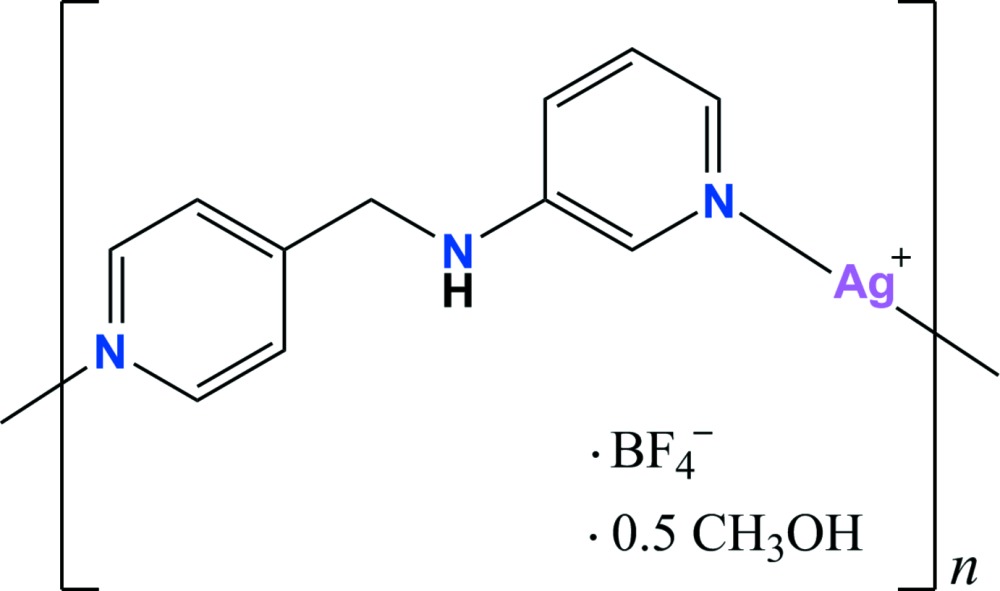


## Structural commentary   

**Figure 1:**
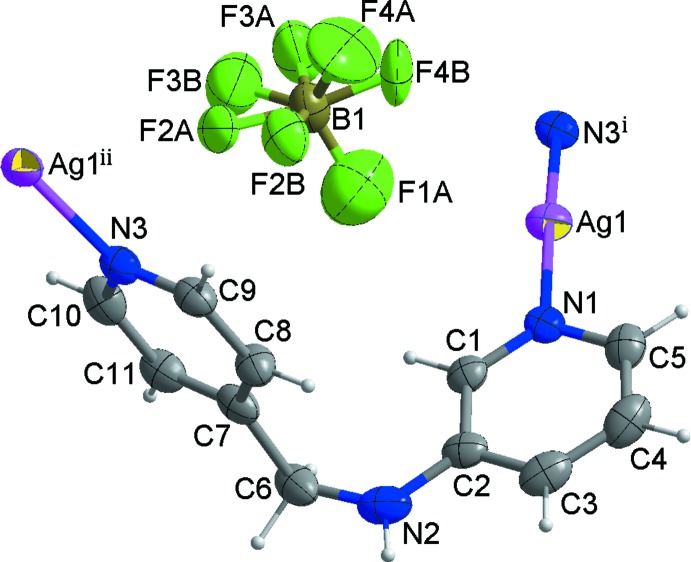
A view of the mol­ecular structure of the title compound with the atom numbering. Displacement ellipsoids are drawn at the 30% probability level. The F atoms of the tetra­fluorido­borate group are disordered over two sets of sites with refined site-occupancy factors of 0.669 (13) (part A) and 0.331 (13) (part B). The disordered methanol solvent mol­ecule is omitted for clarity. [Symmetry codes: (i) − *x* + 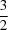, *y* − 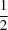, − *z* + 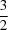; (ii) − *x* + 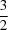, *y* + 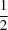, − *z* + 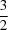.]

**Figure 2:**
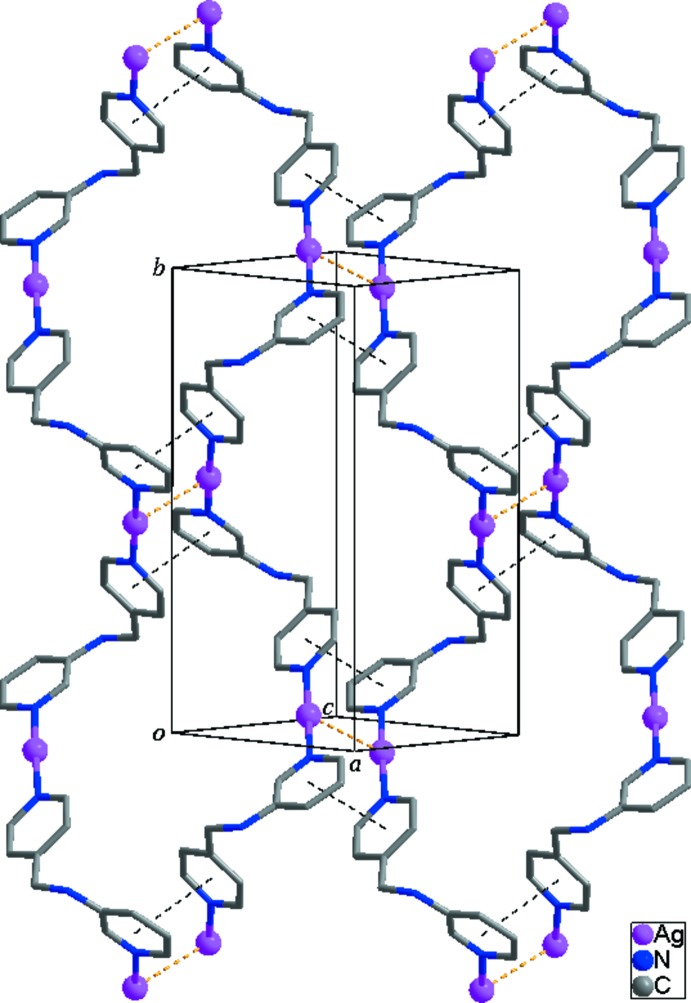
The two-dimensional supra­molecular network formed through Ag⋯Ag (yellow dashed lines) and π–π (black dashed lines) inter­actions. The disordered methanol mol­ecules and tetra­fluorido­borate anions are omitted for clarity.

The mol­ecular components of the title structure are shown in Fig. 1 ▸. The asymmetric unit consists of one Ag^I^ ion, one *L* ligand, one tetra­fluorido­borate anion and one half of a methanol mol­ecule. Each Ag^I^ ion is coordinated by two pyridine N atoms from two symmetry-related ligands in a geometry which is slightly distorted from linear [N1—Ag1—N3 = 174.70 (19)°], forming an infinite helical coordination polymer. The helical chain propagates along [010] (Fig. 2 ▸) with a pitch length of 15.6485 (14) Å, shorter than that [16.7871 (8) Å] of the nitrate-containing Ag^I^ coordination polymer reported by Moon *et al.* (2014 ▸). The two pyridine rings coordinating the Ag^I^ ion are tilted by 13.8 (3)° with respect to each other. The two pyridine rings in the *L* ligand are almost perpendicular, the dihedral angle between their mean planes being 89.34 (15)°.

## Supra­molecular features   

In the crystal structure, symmetry-related right- and left-handed helical chains are arranged alternately through Ag⋯Ag [3.3369 (10) Å] and Ag⋯F inter­actions [Ag1⋯F1*A* = 2.84 (2), Ag1⋯F1*B* = 2.815 (15) and Ag1⋯F4*B* (−*x* + 1, −*y*, −*z* + 1) = 2.879 (10) Å] and π–π inter­actions between the pyridine rings of adjacent helical chains [centroid-to-centroid distance = 3.676 (4) Å], resulting in the formation of a two-dimensional supra­molecular network parallel to the (10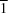) plane (Fig. 2 ▸). Furthermore, several N—H⋯F and C—H⋯F hydrogen bonds (Table 1 ▸) between the helical chains and the anions contribute to stabilization of the crystal structure.

## Database survey   

The non-solvated structures of the silver(I) nitrate and perchlorate complexes of the same ligand have been reported by Zhang *et al.* (2013 ▸). Our group has reported the solvated form of the silver nitrate complex with an *L* ligand (Moon *et al.*, 2014 ▸). These complexes adopt single-stranded helical structures. Our group has also reported the silver tri­fluorido­methane­sulfonate complex with an *L* ligand, which displays a double-stranded helical structure (Lee *et al.*, 2015 ▸).

## Synthesis and crystallization   

The *N*-(pyridin-4-ylmeth­yl)pyridine-3-amine ligand was synthesized according to a literature method (Lee *et al.*, 2013 ▸). X-ray quality single crystals of the title compound were obtained by slow evaporation of a methanol solution of the ligand with AgBF_4_ in the molar ratio 1:1.

## Refinement   

Crystal data, data collection and structure refinement details are summarized in Table 2 ▸. The methanol solvent mol­ecule resides on an inversion centre. Therefore the C12/O12 atoms were refined at the same sites with site occupancy factors of 0.5 using EXYZ/EADP constraints. All H atoms were positioned geometrically and refined using a riding model, with *d*(C—H) = 0.95 Å for C*sp* ^2^—H, 0.88 Å for amine N—H, 0.84 Å for hydroxyl O—H, 0.98 Å for methyl C—H and 0.99 Å for methyl­ene C—H. For all H atoms *U*_iso_(H) = 1.2–1.5*U*_eq_ of the parent atom.

## Supplementary Material

Crystal structure: contains datablock(s) I, New_Global_Publ_Block. DOI: 10.1107/S205698901501837X/cv5498sup1.cif

Structure factors: contains datablock(s) I. DOI: 10.1107/S205698901501837X/cv5498Isup2.hkl

CCDC reference: 1428966

Additional supporting information:  crystallographic information; 3D view; checkCIF report

## Figures and Tables

**Table 1:** Hydrogen-bond geometry (, )

*D*H*A*	*D*H	H*A*	*D* *A*	*D*H*A*
N2H2F2*A* ^i^	0.88	2.10	2.887(9)	148
N2H2F2*B* ^i^	0.88	2.58	3.357(17)	148
C6H6*A*F4*A* ^ii^	0.99	2.39	3.259(12)	146
C10H10F4*A* ^iii^	0.95	2.41	3.318(19)	159
C12H12*B*F1*A*	0.98	2.14	3.08(4)	160

Symmetry codes: (i) 
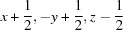
; (ii) 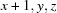; (iii) 
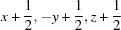
.

**Table 2:** Experimental details

Crystal data	
Chemical formula	[Ag(C_11_H_11_N_3_)](BF_4_)0.5CH_4_O
*M*_r_	395.93
Crystal system, space group	Monoclinic, *P*2_1_/*n*
Temperature (K)	173
*a*, *b*, *c* ()	9.2597(8), 15.6485(14), 10.3574(10)
()	107.185(2)
*V* (^3^)	1433.8(2)
*Z*	4
Radiation type	Mo *K*
(mm^1^)	1.45
Crystal size (mm)	0.50 0.40 0.40

Data collection	
Diffractometer	Bruker SMART CCD area detector
Absorption correction	Multi-scan (*SADABS*; Bruker, 2000 ▸)
*T*_min_, *T*_max_	0.531, 0.595
No. of measured, independent and observed [*I* > 2(*I*)] reflections	8014, 2821, 1883
*R*_int_	0.077
(sin /)_max_ (^1^)	0.617

Refinement	
*R*[*F* ^2^ > 2(*F* ^2^)], *wR*(*F* ^2^), *S*	0.053, 0.167, 1.02
No. of reflections	2821
No. of parameters	227
No. of restraints	31
H-atom treatment	H-atom parameters constrained
_max_, _min_ (e ^3^)	1.18, 0.70

Computer programs: *SMART* and *SAINT-Plus* (Bruker, 2000 ▸), *SHELXS97*, *SHELXL97* and *SHELXTL* (Sheldrick, 2008 ▸) and *DIAMOND* (Brandenburg, 2005 ▸).

## supplementary crystallographic information

### Crystal data

**Table:**

[Ag(C_11_H_11_N_3_)](BF_4_)·0.5CH_4_O	*F*(000) = 780
*M**_r_* = 395.93	*D*_x_ = 1.834 Mg m^−^^3^
Monoclinic, *P*2_1_/*n*	Mo *K*α radiation, λ = 0.71073 Å
Hall symbol: -P 2yn	Cell parameters from 2696 reflections
*a* = 9.2597 (8) Å	θ = 2.4–24.7°
*b* = 15.6485 (14) Å	µ = 1.45 mm^−^^1^
*c* = 10.3574 (10) Å	*T* = 173 K
β = 107.185 (2)°	Block, colorless
*V* = 1433.8 (2) Å^3^	0.50 × 0.40 × 0.40 mm
*Z* = 4	

### Data collection

**Table:**

Bruker SMART CCD area detector diffractometer	2821 independent reflections
Radiation source: fine-focus sealed tube	1883 reflections with *I* > 2σ(*I*)
Graphite monochromator	*R*_int_ = 0.077
φ and ω scans	θ_max_ = 26.0°, θ_min_ = 2.4°
Absorption correction: multi-scan (*SADABS*; Bruker, 2000)	*h* = −10→11
*T*_min_ = 0.531, *T*_max_ = 0.595	*k* = −17→19
8014 measured reflections	*l* = −10→12

### Refinement

**Table:**

Refinement on *F*^2^	Primary atom site location: structure-invariant direct methods
Least-squares matrix: full	Secondary atom site location: difference Fourier map
*R*[*F*^2^ > 2σ(*F*^2^)] = 0.053	Hydrogen site location: inferred from neighbouring sites
*wR*(*F*^2^) = 0.167	H-atom parameters constrained
*S* = 1.02	*w* = 1/[σ^2^(*F*_o_^2^) + (0.1027*P*)^2^] where *P* = (*F*_o_^2^ + 2*F*_c_^2^)/3
2821 reflections	(Δ/σ)_max_ = 0.001
227 parameters	Δρ_max_ = 1.18 e Å^−^^3^
31 restraints	Δρ_min_ = −0.70 e Å^−^^3^

### Special details

**Table:**

Geometry. All e.s.d.'s (except the e.s.d. in the dihedral angle between two l.s. planes) are estimated using the full covariance matrix. The cell e.s.d.'s are taken into account individually in the estimation of e.s.d.'s in distances, angles and torsion angles; correlations between e.s.d.'s in cell parameters are only used when they are defined by crystal symmetry. An approximate (isotropic) treatment of cell e.s.d.'s is used for estimating e.s.d.'s involving l.s. planes.
Refinement. Refinement of *F*^2^ against ALL reflections. The weighted *R*-factor *wR* and goodness of fit *S* are based on *F*^2^, conventional *R*-factors *R* are based on *F*, with *F* set to zero for negative *F*^2^. The threshold expression of *F*^2^ > σ(*F*^2^) is used only for calculating *R*-factors(gt) *etc*. and is not relevant to the choice of reflections for refinement. *R*-factors based on *F*^2^ are statistically about twice as large as those based on *F*, and *R*- factors based on ALL data will be even larger.

### Fractional atomic coordinates and isotropic or equivalent isotropic displacement parameters (Å^2^)

**Table:**

	*x*	*y*	*z*	*U*_iso_*/*U*_eq_	Occ. (<1)
Ag1	0.67175 (6)	−0.04405 (3)	0.54637 (6)	0.0593 (3)	
B1	0.6447 (9)	0.1230 (5)	0.8147 (8)	0.077 (3)	
F1A	0.733 (3)	0.0831 (17)	0.751 (3)	0.217 (10)	0.669 (13)
F2A	0.7295 (8)	0.1786 (5)	0.9050 (9)	0.094 (3)	0.669 (13)
F3A	0.6034 (13)	0.0671 (7)	0.9030 (10)	0.127 (4)	0.669 (13)
F4A	0.5186 (12)	0.1597 (13)	0.7407 (16)	0.197 (8)	0.669 (13)
F1B	0.735 (2)	0.0733 (11)	0.762 (2)	0.072 (5)	0.331 (13)
F2B	0.6706 (19)	0.2035 (7)	0.7697 (18)	0.095 (6)	0.331 (13)
F3B	0.680 (3)	0.1190 (19)	0.9487 (12)	0.143 (9)	0.331 (13)
F4B	0.533 (2)	0.0941 (12)	0.7100 (13)	0.103 (8)	0.331 (13)
N1	0.7799 (6)	0.0402 (3)	0.4442 (6)	0.0510 (13)	
N2	1.1086 (8)	0.1806 (4)	0.5210 (8)	0.0774 (19)	
H2	1.1636	0.2066	0.4765	0.093*	
N3	0.9214 (6)	0.3630 (3)	0.8522 (6)	0.0556 (13)	
C1	0.8958 (7)	0.0881 (4)	0.5113 (7)	0.0515 (15)	
H1	0.9210	0.0901	0.6071	0.062*	
C2	0.9820 (7)	0.1356 (4)	0.4482 (7)	0.0554 (16)	
C3	0.9445 (10)	0.1342 (5)	0.3107 (8)	0.070 (2)	
H3	1.0018	0.1660	0.2650	0.083*	
C4	0.8219 (11)	0.0860 (5)	0.2380 (8)	0.077 (2)	
H4	0.7934	0.0851	0.1420	0.092*	
C5	0.7414 (9)	0.0390 (4)	0.3067 (8)	0.0639 (19)	
H5	0.6580	0.0053	0.2572	0.077*	
C6	1.1541 (8)	0.1869 (4)	0.6647 (10)	0.071 (2)	
H6A	1.2613	0.2049	0.6947	0.085*	
H6B	1.1491	0.1290	0.7015	0.085*	
C7	1.0659 (7)	0.2468 (4)	0.7280 (8)	0.0576 (17)	
C8	0.9678 (8)	0.3085 (4)	0.6527 (8)	0.0617 (18)	
H8	0.9486	0.3119	0.5576	0.074*	
C9	0.8995 (7)	0.3642 (4)	0.7187 (8)	0.0585 (17)	
H9	0.8329	0.4059	0.6666	0.070*	
C10	1.0124 (8)	0.3026 (4)	0.9239 (8)	0.0656 (19)	
H10	1.0274	0.2992	1.0186	0.079*	
C11	1.0858 (8)	0.2446 (4)	0.8627 (8)	0.0621 (18)	
H11	1.1509	0.2028	0.9165	0.074*	
C12	1.022 (2)	0.0409 (10)	0.980 (3)	0.256 (12)	0.50
H12A	1.0264	0.0829	1.0514	0.385*	0.50
H12B	0.9477	0.0594	0.8961	0.385*	0.50
H12C	1.1217	0.0362	0.9658	0.385*	0.50
O12	1.022 (2)	0.0409 (10)	0.980 (3)	0.256 (12)	0.50
H12	1.1101	0.0377	0.9735	0.385*	0.50

### Atomic displacement parameters (Å^2^)

**Table:**

	*U*^11^	*U*^22^	*U*^33^	*U*^12^	*U*^13^	*U*^23^
Ag1	0.0566 (4)	0.0409 (3)	0.0845 (5)	0.0059 (2)	0.0271 (3)	0.0069 (2)
B1	0.081 (6)	0.077 (6)	0.064 (6)	−0.011 (5)	0.010 (5)	−0.004 (5)
F1A	0.237 (13)	0.226 (13)	0.203 (13)	0.004 (9)	0.088 (9)	−0.034 (9)
F2A	0.090 (5)	0.079 (5)	0.120 (8)	−0.025 (4)	0.043 (5)	−0.053 (5)
F3A	0.138 (9)	0.147 (9)	0.090 (7)	−0.053 (7)	0.026 (6)	−0.008 (6)
F4A	0.102 (9)	0.28 (2)	0.173 (12)	0.070 (12)	−0.014 (8)	0.009 (14)
F1B	0.112 (9)	0.053 (7)	0.066 (7)	−0.005 (6)	0.050 (6)	−0.034 (5)
F2B	0.108 (10)	0.057 (7)	0.113 (10)	−0.006 (6)	0.022 (7)	−0.013 (6)
F3B	0.148 (12)	0.155 (13)	0.125 (11)	0.010 (9)	0.037 (9)	−0.024 (9)
F4B	0.154 (19)	0.090 (12)	0.057 (9)	−0.039 (12)	0.015 (10)	−0.029 (8)
N1	0.049 (3)	0.035 (3)	0.069 (4)	0.007 (2)	0.018 (3)	0.001 (2)
N2	0.074 (4)	0.048 (3)	0.127 (6)	0.000 (3)	0.054 (4)	−0.002 (4)
N3	0.051 (3)	0.038 (3)	0.078 (4)	−0.004 (2)	0.018 (3)	−0.004 (3)
C1	0.057 (4)	0.038 (3)	0.062 (4)	0.013 (3)	0.022 (3)	−0.001 (3)
C2	0.058 (4)	0.035 (3)	0.081 (5)	0.011 (3)	0.032 (4)	0.003 (3)
C3	0.092 (6)	0.049 (4)	0.079 (5)	0.025 (4)	0.042 (4)	0.021 (4)
C4	0.115 (7)	0.061 (5)	0.060 (5)	0.025 (5)	0.034 (5)	0.013 (4)
C5	0.073 (5)	0.047 (4)	0.067 (5)	0.015 (3)	0.014 (4)	−0.003 (3)
C6	0.051 (4)	0.045 (4)	0.111 (7)	0.006 (3)	0.014 (4)	−0.019 (4)
C7	0.046 (3)	0.036 (3)	0.085 (5)	−0.004 (3)	0.011 (3)	−0.001 (3)
C8	0.056 (4)	0.048 (4)	0.074 (5)	0.013 (3)	0.009 (3)	−0.008 (3)
C9	0.047 (3)	0.044 (3)	0.078 (5)	0.006 (3)	0.011 (3)	0.001 (3)
C10	0.072 (4)	0.042 (4)	0.074 (5)	−0.008 (3)	0.008 (4)	0.010 (3)
C11	0.054 (4)	0.035 (3)	0.092 (6)	0.002 (3)	0.013 (4)	0.008 (3)
C12	0.145 (14)	0.29 (3)	0.36 (3)	−0.016 (16)	0.106 (15)	−0.01 (2)
O12	0.145 (14)	0.29 (3)	0.36 (3)	−0.016 (16)	0.106 (15)	−0.01 (2)

### Geometric parameters (Å, º)

**Table:**

Ag1—N1	2.118 (5)	C2—C3	1.362 (11)
Ag1—N3^i^	2.121 (5)	C3—C4	1.386 (12)
Ag1—Ag1^ii^	3.3369 (10)	C3—H3	0.9500
B1—F4A	1.324 (8)	C4—C5	1.384 (11)
B1—F3B	1.330 (10)	C4—H4	0.9500
B1—F4B	1.336 (9)	C5—H5	0.9500
B1—F2A	1.348 (11)	C6—C7	1.514 (9)
B1—F1A	1.349 (10)	C6—H6A	0.9900
B1—F1B	1.367 (9)	C6—H6B	0.9900
B1—F2B	1.388 (9)	C7—C11	1.352 (10)
B1—F3A	1.398 (8)	C7—C8	1.396 (9)
N1—C1	1.325 (9)	C8—C9	1.371 (10)
N1—C5	1.363 (10)	C8—H8	0.9500
N2—C2	1.385 (9)	C9—H9	0.9500
N2—C6	1.425 (11)	C10—C11	1.394 (10)
N2—H2	0.8800	C10—H10	0.9500
N3—C10	1.336 (8)	C11—H11	0.9500
N3—C9	1.337 (9)	C12—C12^iv^	1.44 (3)
N3—Ag1^iii^	2.121 (5)	C12—H12A	0.9800
C1—C2	1.388 (9)	C12—H12B	0.9800
C1—H1	0.9500	C12—H12C	0.9800
			
N1—Ag1—N3^i^	174.70 (19)	N1—C1—H1	118.5
N1—Ag1—Ag1^ii^	98.63 (14)	C2—C1—H1	118.5
N3^i^—Ag1—Ag1^ii^	86.18 (14)	C3—C2—N2	119.4 (7)
F4A—B1—F3B	121.9 (14)	C3—C2—C1	118.6 (7)
F4A—B1—F4B	48.5 (9)	N2—C2—C1	121.9 (7)
F3B—B1—F4B	136.5 (18)	C2—C3—C4	119.5 (7)
F4A—B1—F2A	110.8 (11)	C2—C3—H3	120.3
F3B—B1—F2A	52.5 (14)	C4—C3—H3	120.3
F4B—B1—F2A	159.0 (12)	C5—C4—C3	119.3 (7)
F4A—B1—F1A	118.5 (16)	C5—C4—H4	120.4
F3B—B1—F1A	119.4 (17)	C3—C4—H4	120.4
F4B—B1—F1A	83.3 (16)	N1—C5—C4	121.0 (7)
F2A—B1—F1A	109.0 (14)	N1—C5—H5	119.5
F4A—B1—F1B	123.6 (14)	C4—C5—H5	119.5
F3B—B1—F1B	113.5 (15)	N2—C6—C7	117.7 (6)
F4B—B1—F1B	84.3 (15)	N2—C6—H6A	107.9
F2A—B1—F1B	110.2 (10)	C7—C6—H6A	107.9
F1A—B1—F1B	8 (2)	N2—C6—H6B	107.9
F4A—B1—F2B	67.8 (10)	C7—C6—H6B	107.9
F3B—B1—F2B	112.2 (17)	H6A—C6—H6B	107.2
F4B—B1—F2B	101.7 (11)	C11—C7—C8	117.5 (7)
F2A—B1—F2B	61.5 (9)	C11—C7—C6	120.3 (6)
F1A—B1—F2B	93.7 (15)	C8—C7—C6	122.1 (8)
F1B—B1—F2B	101.3 (12)	C9—C8—C7	118.7 (7)
F4A—B1—F3A	106.6 (11)	C9—C8—H8	120.6
F3B—B1—F3A	47.1 (14)	C7—C8—H8	120.6
F4B—B1—F3A	91.4 (10)	N3—C9—C8	123.8 (6)
F2A—B1—F3A	99.5 (7)	N3—C9—H9	118.1
F1A—B1—F3A	110.8 (14)	C8—C9—H9	118.1
F1B—B1—F3A	102.9 (12)	N3—C10—C11	121.3 (7)
F2B—B1—F3A	153.5 (11)	N3—C10—H10	119.3
C1—N1—C5	118.6 (6)	C11—C10—H10	119.3
C1—N1—Ag1	121.3 (5)	C7—C11—C10	121.1 (6)
C5—N1—Ag1	119.8 (5)	C7—C11—H11	119.5
C2—N2—C6	123.0 (6)	C10—C11—H11	119.5
C2—N2—H2	118.5	C12^iv^—C12—H12A	109.5
C6—N2—H2	118.5	C12^iv^—C12—H12B	109.5
C10—N3—C9	117.5 (6)	H12A—C12—H12B	109.5
C10—N3—Ag1^iii^	119.4 (5)	C12^iv^—C12—H12C	109.5
C9—N3—Ag1^iii^	122.9 (4)	H12A—C12—H12C	109.5
N1—C1—C2	123.0 (7)	H12B—C12—H12C	109.5
			
N3^i^—Ag1—N1—C1	−87 (2)	C3—C4—C5—N1	0.7 (10)
Ag1^ii^—Ag1—N1—C1	117.9 (4)	C2—N2—C6—C7	−75.9 (9)
N3^i^—Ag1—N1—C5	86 (2)	N2—C6—C7—C11	168.5 (6)
Ag1^ii^—Ag1—N1—C5	−69.4 (5)	N2—C6—C7—C8	−14.7 (10)
C5—N1—C1—C2	−1.5 (9)	C11—C7—C8—C9	1.2 (10)
Ag1—N1—C1—C2	171.4 (4)	C6—C7—C8—C9	−175.7 (6)
C6—N2—C2—C3	178.6 (6)	C10—N3—C9—C8	−1.6 (10)
C6—N2—C2—C1	−4.6 (10)	Ag1^iii^—N3—C9—C8	175.0 (5)
N1—C1—C2—C3	1.2 (9)	C7—C8—C9—N3	0.0 (10)
N1—C1—C2—N2	−175.6 (6)	C9—N3—C10—C11	2.0 (9)
N2—C2—C3—C4	177.0 (6)	Ag1^iii^—N3—C10—C11	−174.7 (5)
C1—C2—C3—C4	0.2 (10)	C8—C7—C11—C10	−0.8 (10)
C2—C3—C4—C5	−1.0 (11)	C6—C7—C11—C10	176.1 (6)
C1—N1—C5—C4	0.6 (9)	N3—C10—C11—C7	−0.8 (10)
Ag1—N1—C5—C4	−172.4 (5)		

Symmetry codes: (i) −*x*+3/2, *y*−1/2, −*z*+3/2; (ii) −*x*+1, −*y*, −*z*+1; (iii) −*x*+3/2, *y*+1/2, −*z*+3/2; (iv) −*x*+2, −*y*, −*z*+2.

### Hydrogen-bond geometry (Å, º)

**Table:**

*D*—H···*A*	*D*—H	H···*A*	*D*···*A*	*D*—H···*A*
N2—H2···F2*A*^v^	0.88	2.10	2.887 (9)	148
N2—H2···F2*B*^v^	0.88	2.58	3.357 (17)	148
C6—H6*A*···F4*A*^vi^	0.99	2.39	3.259 (12)	146
C10—H10···F4*A*^vii^	0.95	2.41	3.318 (19)	159
C12—H12*B*···F1*A*	0.98	2.14	3.08 (4)	160

Symmetry codes: (v) *x*+1/2, −*y*+1/2, *z*−1/2; (vi) *x*+1, *y*, *z*; (vii) *x*+1/2, −*y*+1/2, *z*+1/2.
